# Transarterial chemoembolization with/without immune checkpoint inhibitors plus tyrosine kinase inhibitors for unresectable hepatocellular carcinoma: a single center, propensity score matching real-world study

**DOI:** 10.1007/s12672-024-00917-1

**Published:** 2024-03-09

**Authors:** Guosheng Yuan, Wenli Li, Mengya Zang, Rong Li, Qi Li, Xiaoyun Hu, Qi Zhang, Wei Huang, Jian Ruan, Huajin Pang, Jinzhang Chen

**Affiliations:** 1 State Key Laboratory of Organ Failure Research, Guangdong Provincial Key Laboratory of Viral Hepatitis Research, Department of Infectious Diseases, Nanfang Hospital, Southern Medical University, Guangzhou, Guangdong 510515 People’s Republic of China; 2 Department of Oncology, Shunde Hospital, Southern Medical University, Shunde, Guangdong 528300 People’s Republic of China; 3 Department of Medical Oncology, School of Medicine, The First Affiliated Hospital, Zhejiang University, Hangzhou, Zhejiang 310003 People’s Republic of China; 4 Division of Vascular and Interventional Radiology, Department of General Surgery, Nanfang Hospital, Southern Medical University, Guangzhou, Guangdong 510515 People’s Republic of China

**Keywords:** Hepatocellular carcinoma, TACE, Immune checkpoint inhibitors, Tyrosine kinase inhibitors, Overall survival

## Abstract

**Objectives:**

To explore the efficacy and safety of Transarterial chemoembolization (TACE) in combination with immune checkpoint inhibitors (ICIs) and tyrosine kinase inhibitors (TKIs) in patients with unresectable hepatocellular carcinoma (uHCC).

**Methods:**

456 patients with HCC receiving either TACE in combination with ICIs and TKIs (combination group, n = 139) or TACE monotherapy (monotherapy group, n = 317) were included from Apr 2016 to Dec 2021 in this retrospective study. We employed propensity score matching (PSM), performed 1:2 optimal pair matching, to balance potential bias.

**Results:**

The mean follow-up time is 24.7 months (95% *CI* 22.6–26.8) for matched patients as of March 2022. After matching, the combination group achieved longer OS and PFS (median OS:21.9 vs. 16.3 months, *P* = 0.022; median PFS: 8.3 vs. 5.1 months, *P* < 0.0001) than TACE monotherapy group. The combination group had better objective response rate (ORR) and disease control rate (DCR) (ORR: 52.5% vs. 32.8%, *P* < 0.001; DCR: 82.7% vs. 59.6%, *P* < 0.001). Subgroup analysis showed that patients who received “TKIs + ICIs” after the first TACE procedure (after TACE group) achieved longer OS than those before the first TACE procedure (before TACE group) (26.8 vs. 19.2 months, *P* = 0.011). Adverse events were consistent with previous studies of TACE-related trials.

**Conclusions:**

TACE plus TKIs and ICIs appeared to deliver longer PFS and OS in HCC patients than TACE monotherapy. “TKIs + ICIs” co-treatment within 3 months after the first TACE procedure might be a better medication strategy.

**Supplementary Information:**

The online version contains supplementary material available at 10.1007/s12672-024-00917-1.

## Introduction

Liver cancer (LC) is one of the most prevalent causes of cancer-related death globally, especially hepatocellular carcinoma (HCC) associated with hepatitis B virus in Asia. [1] Although increasing rates of screening and surveillance for LC across most countries, more than 70% of patients are still diagnosed as intermediate or advanced-stage disease, leading to poor prognosis. [2–4]. TACE is widely used in intermediate HCC globally and in intermediate-advanced HCC domestically. [5–8] However, Transarterial chemoembolization (TACE) has not been universally successful in controlling tumor growth, recurrence and metastasis due to the high rate of incomplete embolization and embolization-induced changes in the tumor microenvironment (TME). [9, 10]

There are some rationales about TACE combining with immune checkpoint inhibitors (ICIs) and tyrosine kinase inhibitors (TKIs). Embolization-induced hypoxia after TACE can lead to upregulation of vascular endothelial growth factor (VEGF), which might contribute to revascularization, making it the theoretical basis of using anti-angiogenesis in the combination therapy. [11, 12] The efficacy of TACE in combination with anti-angiogenesis drugs in HCC has been proven in preclinical and clinical trials. [13–15] In addition, TACE results in necrosis of the tumor tissue and releases tumor antigens which may promote tumor-specific immune responses. [16] It is reported that intra-tumoral exhausted effector cells, such as CD8 + /PD-1 + T cells and T regulatory cells (CD4 + /FOXP3 +) would become lower after TACE, which may transform an immuno-suppressive microenvironment into an immune supportive setting to enhance the response of ICIs. [17] Therefore, TACE combined with TKI and ICI is theoretically reasonable. To our knowledge, few data of prospective clinical trials are published about this regimen. Herein, more research is needed on the efficacy and safety of this triple combination therapy.

Therefore, we aimed to further validate the better efficacy with acceptable safety of TACE with ICI plus TKI than TACE monotherapy in this retrospective, propensity score matching (PSM) cohort of HCC patients in the real-world study.

## Materials and methods

### Patients

**Fig. 1:**
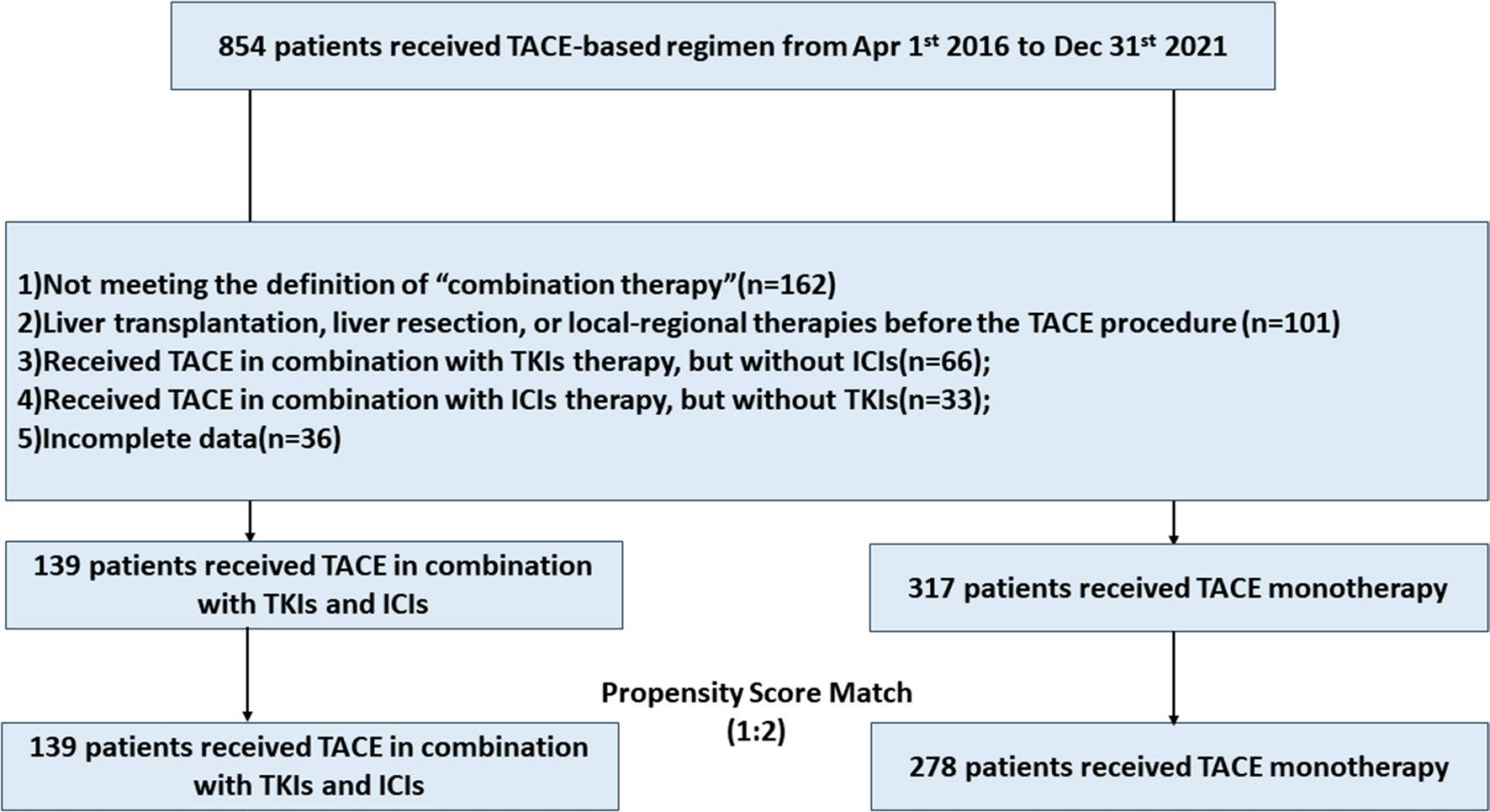
The flowchart of patients’ selection

This retrospective study reviewed data of 854 unresectable HCC patients receiving TACE-based therapy from April 2016 to December 2021 (Fig. 1).

The inclusion criteria were as follows: (1) histologically, cytologically or clinically confirmed diagnosis of HCC according to the European Association for the Study of the Liver (EASL) criteria[5]; (2) at least 1 measurable lesion according to modified Response Evaluation Criteria in Solid Tumors (mRECIST, version 1.1); (3) with BCLC stage B or C; (4)received TACE or TACE + TKIs + ICIs as the first-line treatment, (5) received at least one cycle of TACE based therapy.

The exclusion criteria were as follows: (1) with Child–Pugh C liver function; (2) Eastern Cooperative Oncology Group (ECOG) score of ≥ 3 points; (3) liver transplantation, liver resection, or local–regional therapies before the TACE procedure; (4) currently had or had a history of malignant tumors in addition to HCC; (5) incomplete data;(6) not meeting the definition of combination therapy.

In the end, a total of 456 patients were selected for the study. Patients were assigned into 2 groups according to different treatment strategies: TACE monotherapy group and TACE in combination with TKIs and ICIs therapy group (combination group) (Fig. 1). Combination therapy was defined as treatment with TKIs and ICIs within 1 month before, or within 3 months after the first TACE procedure, while the interval between TKIs and ICIs must be within 1 week in the TACE + TKIs + ICIs group. To reduce the impact of potential confounders, we employed propensity score matching, performed 1:2 optimal pair matching to balance the covariates between the treatment groups (Table 1).

**Table 1:** Baseline characters of patients before and after PSM

Variables	Unmatched			Matched		
	TACE, N = 317^1^	TACE + TKIs + ICIs, N = 139^1^	p-value^2^	TACE, N = 278^1^	TACE + TKIs + ICIs, N = 139^1^	p-value^2^
Age(y)			0.708			0.942
Median (IQR)	59 (51, 66)	59 (50, 67)		58 (50, 67)	59 (50, 67)	
Gender			0.144			0.315
Male	290 (91.48%)	121 (87.05%)		251 (90.29%)	121 (87.05%)	
Female	27 (8.52%)	18 (12.95%)		27 (9.71%)	18 (12.95%)	
BCLC			0.470			0.879
B	215 (67.82%)	99 (71.22%)		196 (70.50%)	99 (71.22%)	
C	102 (32.18%)	40 (28.78%)		82 (29.50%)	40 (28.78%)	
ECOG			0.408			0.776
0	259 (81.70%)	118 (84.89%)		233 (83.81%)	118 (84.89%)	
1	58 (18.30%)	21 (15.11%)		45 (16.19%)	21 (15.11%)	
Tumor Number			0.709			0.554
≥ 3 nodules	249 (78.55%)	107 (76.98%)		221 (79.50%)	107 (76.98%)	
< 3 nodules	68 (21.45%)	32 (23.02%)		57 (20.50%)	32 (23.02%)	
Tumor Size(cm)			0.676			0.512
< 7	109 (34.38%)	45 (32.37%)		99 (35.61%)	45 (32.37%)	
≥ 7	208 (65.62%)	94 (67.63%)		179 (64.39%)	94 (67.63%)	
Extrahepatic metastasis			0.228			0.926
absent	249 (78.55%)	116 (83.45%)		231 (83.09%)	116 (83.45%)	
present	68 (21.45%)	23 (16.55%)		47 (16.91%)	23 (16.55%)	
PVTT			0.698			0.869
absent	241 (76.03%)	108 (77.70%)		214 (76.98%)	108 (77.70%)	
present	76 (23.97%)	31 (22.30%)		64 (23.02%)	31 (22.30%)	
AFP (ng/ml)			0.608			0.874
< 400	242 (76.34%)	103 (74.10%)		208 (74.82%)	103 (74.10%)	
≥ 400	75 (23.66%)	36 (25.90%)		70 (25.18%)	36 (25.90%)	
Ascites			0.791			0.731
absent	252 (79.50%)	112 (80.58%)		220 (79.14%)	112 (80.58%)	
present	65 (20.50%)	27 (19.42%)		58 (20.86%)	27 (19.42%)	
Child–Pugh			0.903			0.922
A	270 (85.17%)	119 (85.61%)		237 (85.25%)	119 (85.61%)	
B	47 (14.83%)	20 (14.39%)		41 (14.75%)	20 (14.39%)	
WBC(*10^9^)			0.746			0.878
Median (IQR)	5.21 (3.79, 6.67)	4.93 (3.95, 6.57)		5.23 (3.71, 6.64)	4.93 (3.95, 6.57)	
NEU(*10^9^)			0.684			0.880
Median (IQR)	2.91 (1.99, 4.05)	2.73 (2.00, 3.90)		2.88 (1.98, 3.99)	2.73 (2.00, 3.90)	
PLT(*10^9^)			0.451			0.515
Median (IQR)	141 (89, 208)	152 (101, 197)		141 (88, 214)	152 (101, 197)	
ALT(U/L)			0.155			0.151
Median (IQR)	29 (21, 43)	32 (21, 48)		29 (21, 42)	32 (21, 48)	
AST(U/L)			0.700			0.836
Median (IQR)	34 (24, 49)	32 (24, 47)		33 (24, 49)	32 (24, 47)	
TBIL (µmol/L)			0.738			0.683
Median (IQR)	13 (9, 20)	13 (9, 18)		13 (9, 21)	13 (9, 18)	
ALB(g/L)			0.889			0.996
Median (IQR)	37.5 (33.7, 41.0)	37.2 (34.0, 41.3)		37.6 (34.0, 40.9)	37.2 (34.0, 41.3)	
PT(s)			0.478			0.282
Median (IQR)	11.90 (11.10, 12.80)	11.70 (11.00, 12.80)		11.90 (11.20, 12.90)	11.70 (11.00, 12.80)	
TACE times			0.386			0.751
Median (IQR)	2.00 (1.00, 4.00)	2.00 (1.00, 3.00)		2.00 (1.00, 4.00)	2.00 (1.00, 3.00)	
HBsAg			0.732			0.853
Positive	258 (81.39%)	115 (82.73%)		232 (83.45%)	115 (82.73%)	
Negative	59 (18.61%)	24 (17.27%)		46 (16.55%)	24 (17.27%)	
HBsAb			0.714			> 0.999
Negative	279 (88.01%)	124 (89.21%)		248 (89.21%)	124 (89.21%)	
Positive	38 (11.99%)	15 (10.79%)		30 (10.79%)	15 (10.79%)	
HBeAg			0.558			0.670
Negative	282 (88.96%)	121 (87.05%)		246 (88.49%)	121 (87.05%)	
Positive	35 (11.04%)	18 (12.95%)		32 (11.51%)	18 (12.95%)	
HBeAb			0.565			0.781
Positive	139 (43.85%)	65 (46.76%)		126 (45.32%)	65 (46.76%)	
Negative	178 (56.15%)	74 (53.24%)		152 (54.68%)	74 (53.24%)	
HBcAg			0.277			0.613
Positive	215 (67.82%)	87 (62.59%)		181 (65.11%)	87 (62.59%)	
negative	102 (32.18%)	52 (37.41%)		97 (34.89%)	52 (37.41%)	
HBVDNA			0.390			0.573
Positive	121 (38.17%)	59 (42.45%)		110 (39.57%)	59 (42.45%)	
Negative	196 (61.83%)	80 (57.55%)		168 (60.43%)	80 (57.55%)	
HCVRNA			0.786			> 0.999
Negative	305 (96.21%)	135 (97.12%)		269 (96.76%)	135 (97.12%)	
Positive	12 (3.79%)	4 (2.88%)		9 (3.24%)	4 (2.88%)	

TACE, transarterial chemoembolization; TKIs, tyrosine kinase inhibitors; ICIs, Immune checkpoint inhibitors; ECOG, Eastern Cooperative Oncology Group; AFP, α-fetoprotein; WBC, white blood cell; NEU, neutrophils; ALT, alanine aminotransferase; AST, aspartate aminotransferase; TBIL, total bilirubin; ALB, albumin; PT, prothrombin time; PLT, platelet count; HBsAg, hepatitis B surface antigen; Anti-HBs, hepatitis B s antibody; HBeAg, hepatitis B e antigen; Anti-HBe, hepatitis B e antibody; Anti-HBc, hepatitis B core antibody; HBVDNA, DNA of hepatitis B virus; HCVRNA, RNA of hepatitis C virus;

^1^n (%),^2^Wilcoxon rank sum test; Pearson's Chi-squared test; Fisher's exact test

This study was designed and performed according to the Declaration of Helsinki and was approved by the Medical Ethics Committee of Nanfang Hospital, Southern Medical University, and written, informed consent was obtained from each patient to retrospectively review and report on their medical records.

### Treatment protocol

#### TACE procedures

The TACE protocol was performed as the previous study stated. Briefly, an emulsion mixed with lipiodol (5–20 mL) and adriamycin (20–50 mg) and subsequent 500–700 μm absorbable gelatin sponge particles were injected into the tumor feeding artery for chemoembolization.

#### The administration of TKIs

TKIs was given at a metronomic oral dosage (400 mg, twice daily, for sorafenib; 8 mg, daily, for lenvatinib; 80 mg, daily, for Regorafenib; 250 mg, daily, for Apatinib).

#### The administration of ICIs

Toripalimab was given at a fixed dose of 240 mg; Camrelizumab, Sintilimab and Tislelizumab was given at a fixed dose of 200 mg; Atezolizumab was given at a fixed dose of 1200 mg; Pembrolizumab was given at 2 mg/kg body weight; Nivolumab was given at 3 mg/kg body weight; All ICIs were given every 3 weeks intravenously.

The grading of adverse events related to TKIs, and ICIs were conducted according to the National Cancer Institute Common Terminology Criteria for Adverse Events (NCI-CTCAE 5.0). If adverse events ≥ 3 grade (defined as serious adverse events, SAEs) were observed, dosages of TKIs and ICIs were reduced, suspended, or discontinued.

### Study endpoints

The primary endpoints of this study were OS (defined as the interval from treatment initiation (TACE or the first dose of TKIs/ICIs administration) to patient death or the last follow-up) and progression free survival (PFS) (defined as the time from treatment initiation to the first reported disease progression or death from any cause). The secondary endpoints were objective response rate (ORR) and disease control rate (DCR), according to the tumor response evaluated by two experienced radiologists using mRECIST. The ORR was defined as the percent of the total number of patients who had a complete response (CR) and partial response (PR), while DCR was defined as the percent of the total number of patients who had a CR, PR and stable disease (SD). Adverse events (AEs) were graded according to NCI-CTCAE 5.0. In addition, we had analyzed the predictors of affecting OS in the study. Whether the timing of TACE in combination with TKIs and ICIs affects OS was further explored in the TACE + TKIs + ICIs group.

### Statistical analysis

To address the imbalance of potential confounders between two groups, we performed 1:2 optimal pair matching, propensity scores were calculated using logistic regression, taking into account the aforementioned demographic and clinical characteristic, including Age, Gender, BCLC, ECOG, Tumor Number, Tumor Size, Extrahepatic metastasis, PVTT, AFP, Ascites, Child–Pugh, WBC, NEU, ALT, AST, TBIL, ALB, PT, PLT, HBsAg, HBsAb, HBeAg, HBeAb, HBcAg, HBVDNA, and HCV, TACE time for both groups.

The patient characteristics were summarized using median with IQR for continuous variables and frequencies with proportions for categorical variables. Student’s t-test or Mann–Whitney U test was used to analyze continuous variables. Chi-squared test or Fisher exact test was applied to analyze categorical variables. The difference in PFS and OS between the two groups were compared with the use of a log-rank test. The survival curves were plotted by the method of Kaplan–Meier.

Cox proportion hazards model was used for multivariable analyses on the propensity-matched sample. Forest plots were used to display these data.

A two-tailed P-value of < 0.05 was considered statistically significant. All the above statistical analyses were performed using R (version 4.2.2; R Project for Statistical Computing, http://

www.r-project.org), MSTATA software and SPSS (version 25.0; IBM, SPSS).

## Results

### Patient baseline characteristics

A total of 456 patients were included in the final study: 317 (69.5%) patients received TACE monotherapy (TACE monotherapy group), and 139 (30.5%) patients received TACE in combination with TKIs and ICIs therapy (combination group), respectively. The mean follow-up time is 17.1 months (95% *CI* 16.3–17.9 months) and the mean number of TACE sessions were 2.7 (95% *CI* 2.5–2.9) for all patients as of March 2022. In the TACE monotherapy group, 102 (32.2%) patients were BCLC stage C, 259 (81.7%) had an ECOG performance score of 0, 249 (78.5%) had ≥ 3 tumor nodules, 76 (24.0%) were embolus existed; In the TACE + TKIs + ICIs group, 40 (28.8%) patients were BCLC stage C, 118 (84.9%) had an ECOG performance score of 0, 107 (77.0%) had ≥ 3 tumor nodules, 31 (22.3%) were embolus existed. There were no significant differences in baseline parameters between two groups both before and after matching (Table 1).

### Efficacy

**Fig. 2:**
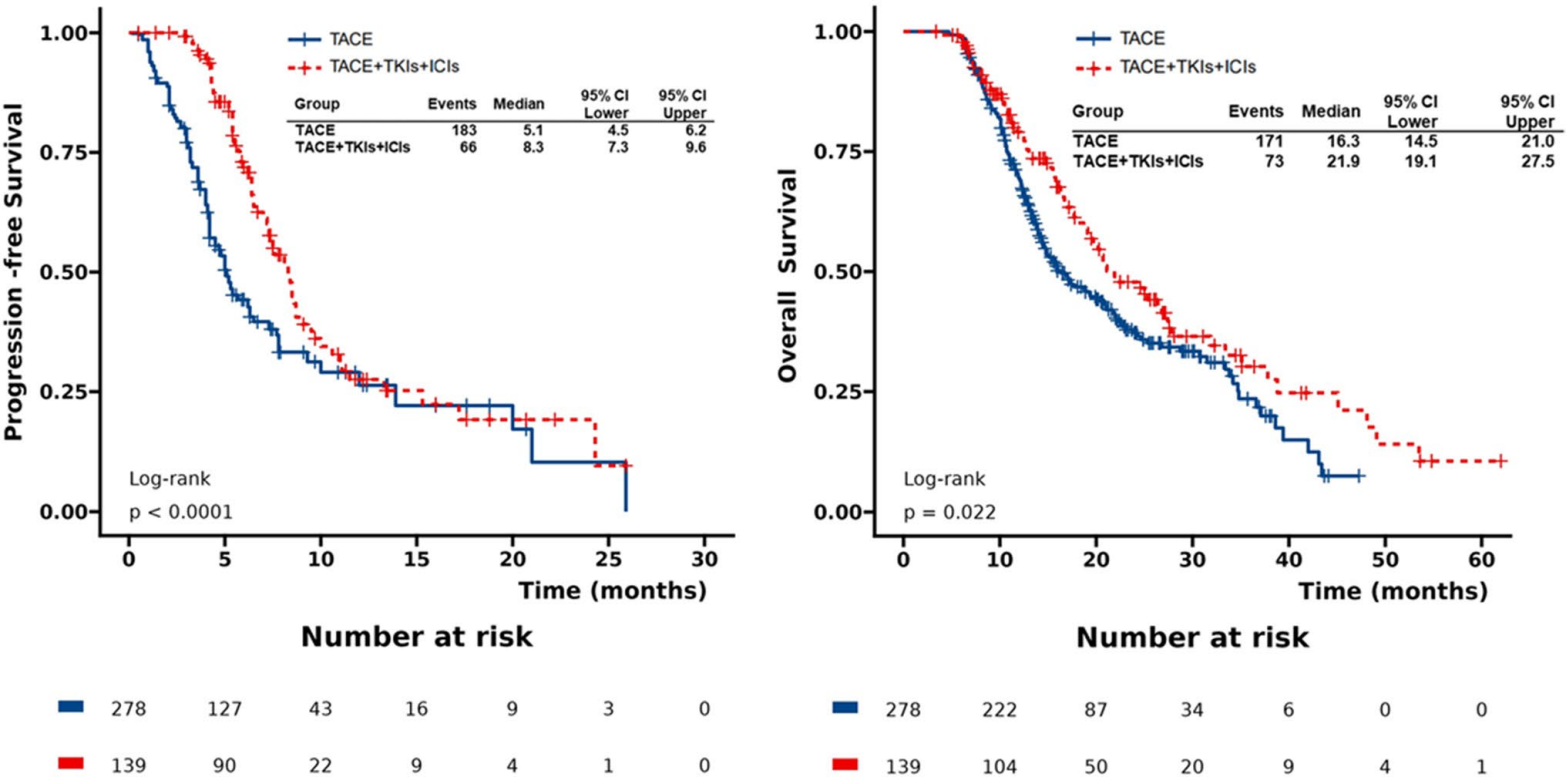
Kaplan–Meier plots of progression-free survival and overall survival after PSM

After matching, the median OS (mOS) and median PFS (mPFS) were both longer in TACE + TKIs + ICIs group than TACE alone with significant differences between the two group. The mOS were 21.9 months (95% CI 19.1–27.5 months) and 16.3 months (95% CI 14.5–21.0 months), respectively (*P* = 0.022). The mPFS were 8.3 months (95% CI 7.3–9.3 months) and 5.1 months (95% CI 4.5–6.2 months, respectively (*P* < 0.0001) (Fig. 2). More details about final status of patients are shown in Additional file 1: Table S2.

**Figure3:**
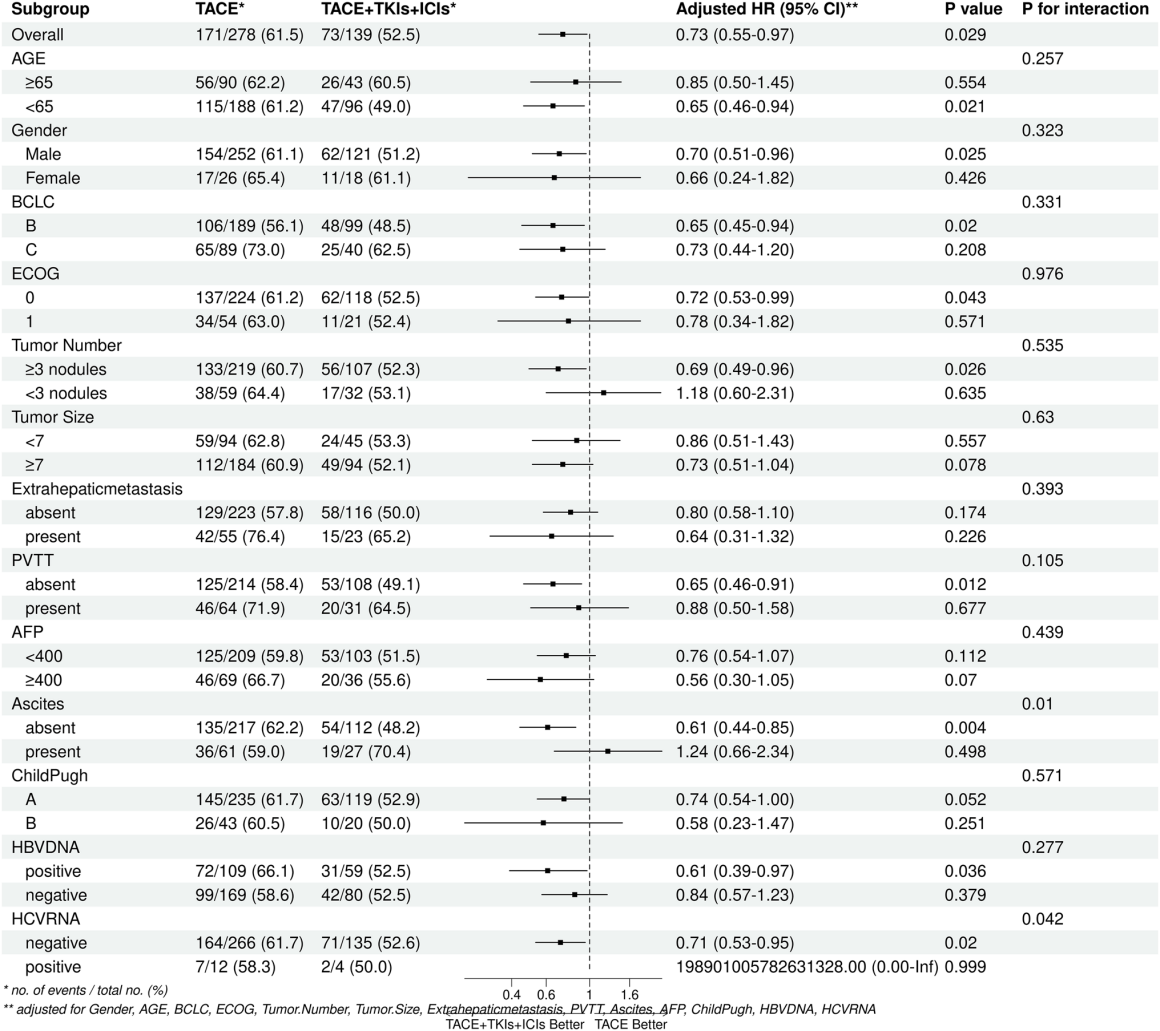
Subgroup analysis of mOS

We used multivariableCox proportionalhazards model showed that combination therapy was the independent positive prognostic indicator for OS in the matched cohorts (hazard ratio [HR], 0.72,95% CI,0.54–0.95,*P* = 0.022 in univariable analysis; HR, 0.73,95% CI, 0.55–0.97,*P* = 0.032 in multivariable analysis), while Tumor Number is also another independent predictors of OS. (Table 2)Subgroup analysis of OS showed that the combination group had a trend persisted on better clinical benefits compared to the monotherapy group (Fig. 3). Additionally, we conducted Logistic regression analysis using progressive disease(PD) as a binary variable and found that the PD rate advantage of the TACE + TKIs + ICIs group is about 0.69 times that of the TACE group in the overall population (OR = 0.69, 95% CI 0.46–1.04, *P* = 0.079) (Additional file 1 Figure S1). At different subgroup levels, the OR values are always less than 1, implying a trend of lower rate of PD in the TACE + TKIs + ICIs than that of the TACE group.

**Table 2:** Predictors of overall survival after matching

	Univariable					Multivariable				
Characteristic	N	Event N	HR^1^	95% CI^1^	p-value	N	Event N	HR^1^	95% CI^1^	p-value
Gender										
Male	373	216	–	–		373	216	–	–	
Female	44	28	1.02	0.68, 1.51	0.935	44	28	1.25	0.81, 1.91	0.312
Age	417	244	1.00	0.99, 1.01	0.948	417	244	1.00	0.99, 1.01	0.745
BCLC										
B	288	154	–	–		288	154	–	–	
C	129	90	1.74	1.34, 2.27	< 0.001	129	90	1.25	0.72, 2.17	0.428
ECOG										
0	342	199	–	–		342	199	–	–	
1	75	45	1.11	0.80, 1.53	0.546	75	45	1.09	0.78, 1.54	0.608
Tumor number										
≥ 3 nodules	326	189	–	–		326	189	–	–	
< 3 nodules	91	55	1.43	1.06, 1.93	0.020	91	55	1.43	1.03, 1.98	0.031
Tumor Size										
< 7	139	83	–	–		139	83	–	–	
≥ 7	278	161	1.13	0.86, 1.47	0.385	278	161	1.01	0.76, 1.34	0.962
Extrahepatic metastasis										
Absent	339	187	–	–		339	187	–	–	
Present	78	57	1.64	1.22, 2.21	0.001	78	57	1.49	1.00, 2.21	0.051
PVTT										
Absent	322	178	–	–		322	178	–	–	
Present	95	66	1.63	1.23, 2.17	< 0.001	95	66	1.15	0.70, 1.91	0.579
AFP										
< 400	312	178	–	–		312	178	–	–	
≥ 400	105	66	1.32	1.00, 1.76	0.053	105	66	1.17	0.87, 1.59	0.302
Child Pugh										
A	354	208	–	–		354	208	–	–	
B	63	36	0.93	0.65, 1.32	0.682	63	36	0.97	0.67, 1.41	0.881
Ascites										
Absent	329	189	–	–		329	189	–	–	
Present	88	55	1.14	0.84, 1.53	0.410	88	55	1.07	0.78, 1.48	0.656
HBVDNA										
Positive	168	103	–	–		168	103	–	–	
Negative	249	141	0.76	0.59, 0.99	0.038	249	141	0.80	0.60, 1.05	0.112
HCVRNA										
Negative	401	235	–	–		401	235	–	–	
Positive	16	9	1.24	0.64, 2.42	0.523	16	9	1.22	0.61, 2.47	0.571
Group										
TACE	278	171	–	–		278	171	–	–	
TACE + TKIs + ICIs	139	73	0.72	0.54, 0.95	0.022	139	73	0.73	0.55, 0.97	0.032

^1^HR = Hazard Ratio, CI = Confidence Interval

In the TACE monotherapy group, 6 (1.9%) patients had complete response (CR), 98 (30.9%) patients achieved PR, and 85 (26.8%) patients had SD, resulting in an ORR of 32.8% and DCR of 59.6%. In the TACE + TKIs + ICIs group, 9 (6.5) and 64 (46.0%) participants achieved CR and PR, respectively, with 42 (30.2%) patients experiencing SD and 24 (17.3%) experiencing PD. The ORR was 52.5%, and the DCR was 82.7% for this treatment group (*P* = 0.001 for ORR comparison; *P* < 0.001 for DCR comparison) (Table 3).

**Table 3:** Tumor responses

Tumor response, n (%)	TACE (n = 317)	TACE + TKIs + ICIs (n = 139)
Complete response (CR)	6 (1.9)	9 (6.5)
Partial response (PR)	98 (30.9)	64 (46.0)
Stable disease (SD)	85 (26.8)	42 (30.2)
Progressive disease (PD)	128 (40.4)	24 (17.3)
ORR (CR + PR)^*^	104 (32.8)	73 (52.5)
DCR (CR + PR + SD)^#^	189 (59.6)	115 (82.7)

DCR, disease control rate; ORR, objective response rate

^*^Pearson χ^2^ = 15.807, *P* < 0.001 (ORR comparison between the 2 groups)

^#^Pearson χ^2^ = 23.228, *P* < 0.001 (DCR comparison between the 2 groups)

multivariable.

### Safety

Totally 248 patients (78.2%) in the TACE monotherapy group and 112 (80.6%) in the TACE + TKIs + ICIs group, experienced at least one AEs of any grade. The most common treatment-related AEs (TRAEs) were pain (68.8%), fever (51.4%), and thrombocytopenia (49.8%) in the TACE monotherapy group; Fatigue (62.6%), Aspartate aminotransferase increased (61.9%), and proteinuria (59.7%) in the TACE + TKIs + ICIs group.

The TRAEs ≥ 3 grade led to dose reduction in 36 (11.4%) patients and treatment termination in 2 (0.6%) patients in the TACE monotherapy group, and 30 (21.6%) patients and 8 (5.8%) patients in the TACE + TKIs + ICIs group. AEs in all patients were effectively controlled by symptomatic treatment or reduction in drug dose or discontinuation, and there were no treatment-related deaths (Table 4).

**Table 4:** Treatment-related adverse events

Effects	TACE (n = 317)	TACE + TKIs + ICIs (n = 139)	P value
Alanine aminotransferase increased	134 (42.3)	79 (56.8)	0.004
Alimentary tract hemorrhage	16 (5.0)	13 (9.4)	0.083
Aspartate aminotransferase increased	143 (45.1)	86 (61.9)	0.001
Diarrhea	92 (29.0)	51 (36.7)	0.104
Fatigue	114 (36.0)	87 (62.6)	< 0.001
Fever	163 (51.4)	77 (16.5)	0.434
Gingivitis	62 (19.6)	23 (16.5)	0.447
Hand-foot syndrome	41 (12.9)	72 (51.8)	< 0.001
Hematuria	34 (10.7)	19 (13.7)	0.367
Hyperbilirubinemia	22 (6.9)	42 (30.2)	< 0.001
Hypertension	17 (5.4)	53 (38.1)	< 0.001
Hyperthyroidism^Δ^	8 (2.5)	5 (3.6)	0.547
Hypothyroidism^Δ^	4 (1.3)	6 (4.3)	0.074
Nausea	117 (36.9)	67 (48.2)	0.024
Pain	218 (68.8)	71 (51.1)	< 0.001
Proteinuria	14 (4.4)	83 (59.7)	< 0.001
Rash	76 (24.0)	53 (38.1)	0.002
Thrombocytopenia	158 (49.8)	82 (59.0)	0.072
Weight loss	37 (11.7)	71 (51.1)	< 0.001
Grade ≥ 3	36 (11.4)	30 (21.6)	0.007
Dose reduction	2 (0.6)	8 (5.8)	0.002

Δ: Fisher's exact test, others used χ^2^ tests

### Subgroup analysis of “before TACE group” and “after TACE group”

**Fig. 4:**
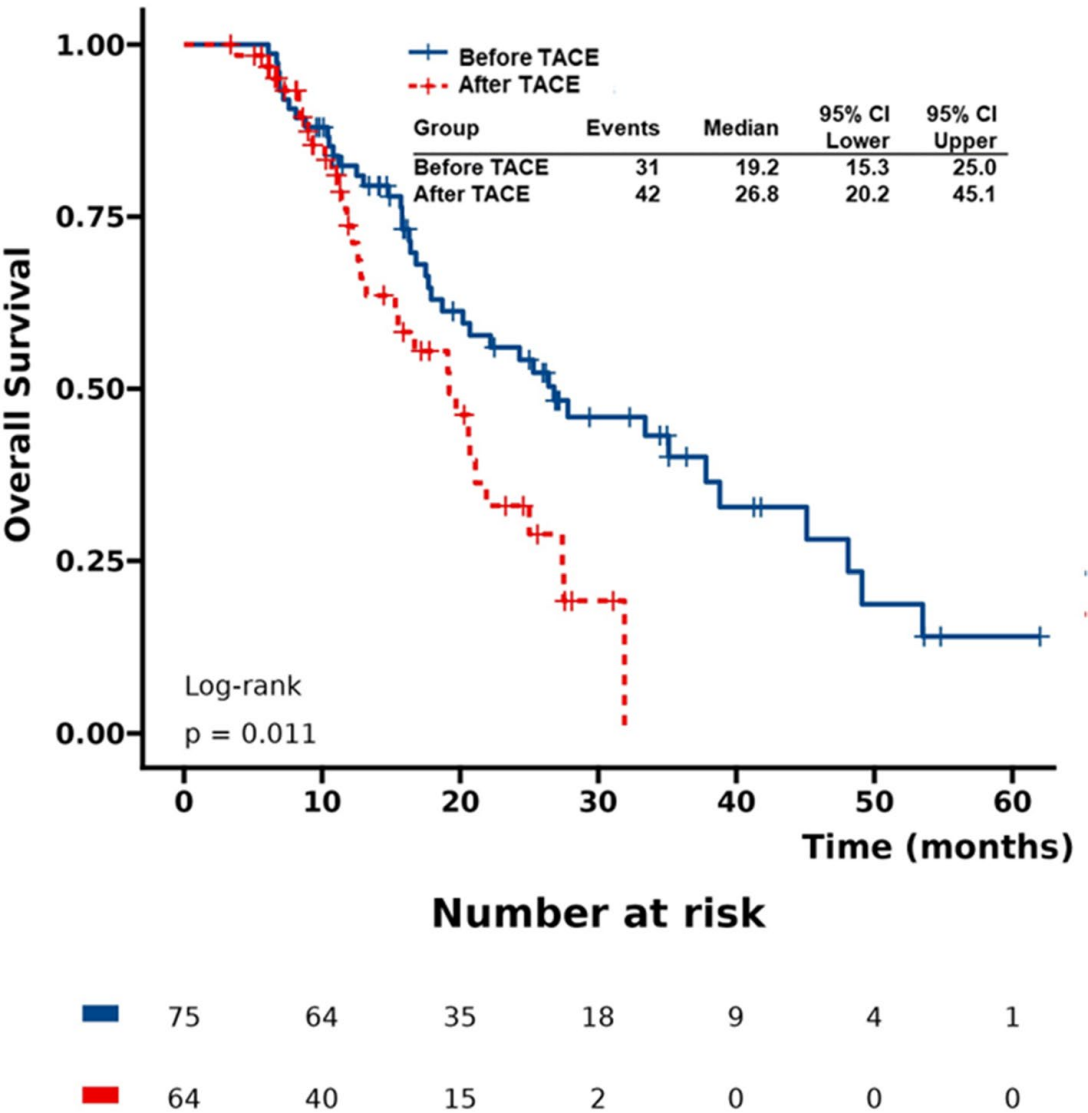
Kaplan–Meier plots of overall survival between “after TACE” and “before TACE” group

To determine the timing of TACE in combination with TKIs and ICIs, the mOS was compared between the “before TACE group” (the “TKIs + ICIs” co-treatment was given within 1 month before the first TACE procedure) and the “after TACE group” (the “TKIs + ICIs” co-treatment was given within 3 months after the first TACE procedure). According to Figure S1 in Additional file 1, the mOS was 19.2 months (95% CI 15.3–25.0 months) in the “before TACE group” and 26.8 months (95% CI 20.2–45.1 months) in the “after TACE group” (*P* = 0.011).(Fig. 4).

## Discussion

Our study showed that TACE in combination with TKIs and ICIs achieved the higher mOS (21.9 months, 95% CI 17.2–26.6 months) and mPFS (8.3 months, 95% CI 6.0–7.4 months) when compared to TACE alone. Subgroup analyses showed that the mOS was 19.2 months (95% CI 14.3–24.1 months) in the “before TACE group” and 26.8 months (95% CI 15.7–37.9 months) in the “after TACE group” (*P* = 0.011). No worse AEs were observed in the present study compared with previously reported clinical trials. [18–20] Most AEs were easily managed and controlled with mild-to-moderate severity, consistent with the results of other previous studies. This combination regimen promises to be a potential first-line option for patients with advanced HCC.

Currently, immunotherapy has approved for the treatment in HCC. “A + T” has been recommended as the first-line treatment in andvanced HCC after IMbrave150 trial published. Compared to updated data of IMbrave150 and Oriental-32, our study seemed to delivery more promising mOS (21.9 vs 19.2 compared IMbrave150, 21.9 vs 10.4 compared Orient-32) and mPFS (8.3 vs 6.9 compared IMbrave150, 8.3 vs 6.9 compared Orient-32). [21, 22] It probably means that combining TACE with TKIs + ICIs improves mOS and mPFS of patients with intermediate-advanced HCC. This has also been validated in real-world studies. Median OS and PFS of combination group were 19.2 months [95%CI (16.1–27.3)] and 9.5 months [95%CI (8.4–11.0)] in CHANCE001 trial, 24.1 months [95%CI 20.0-NR] and 13.5 months [95%CI 9.0–18.0] in CHANCE2211 trial, respectively. [23, 24] Our study demonstrated considerable efficacy, especially in “after TACE” group. However, the ORR of immunotherapy in HCC patients is 15–30%. Its efficacy is affected by a variety of factors, such as liver function status, BCLC staging, baseline tumor burden, etiology, etc. [25, 26] How to improve the efficacy of immunotherapy is still a problem for clinicians. The OS benefit with atezolizumab plus bevacizumab vs. sorafenib was generally consistent across the patient subgroups in IMbrave150 trial, except in patients who had non-viral etiology of HCC (HR for death 1.05; 95% CI 0.68–1.63). [25, 27] Immune therapy did not improve survival in patients with non-viral HCC reported in a meta-analysis, which showed that non-viral HCC, might be less responsive to immunotherapy. That was visibly observed in HCC caused by Non-Alcoholic SteatoHeaptitis(NASH-HCC) probably owing to NASH-related aberrant T cell activation causing tissue damage and impaired immune surveillance. [25, 28]While the synergistic antitumor effects underlying TACE plus ICIs and TKIs was verified and could possibly be explained as: (1) TACE causes necrosis and disintegration of the tumor cells, which results in releasing a large number of tumor antigens, increasing the expression of programmed cell death protein-1 (PD-1) and programmed cell death protein ligand-1 (PD-L1), and improving tumor recognition [12, 17]; (2) TACE could alter the TME of HCC by producing local inflammation, promoting lymphocyte infiltration, and activating the immune system, which may improve the overall anticancer effect of ICIs [16, 29]; (3) TKIs could antagonize the effect of TACE-induced angiogenesis by blocking the VEGF signaling pathway and enhance the efficacy of ICIs by reversing VEGF-mediated immunosuppression [30–32]. These proposed mechanisms suggest that the combination of TACE, TKIs and ICIs might produce synergistic anti-HCC effects and therefore improve the OS and PFS of the patients.

In the subgroup analysis, “After TACE group” delivered more promising result of OS compared “Before TACE group”. One possible explanation for prolong mOS is that the efficacy of TACE, tumor antigens releasing and TME altering, are time-consuming [33], possibly due to the worsened hypoxia and immunosuppressive TME of residual tumor tissue following TACE [9, 32]. At this consuming moment, TKIs are needed to alleviate hypoxia and immunosuppressive TME through vascular normalization so that the inflammatory environment suitable for T-cell responses could be maintained, which improves the therapeutic effectiveness of ICIs [31]. In addition, previous studies have found a significant infiltration of CD8 + T cells after TACE, indicating an immunological enhancement in the body [12, 34, 35]. LAUCH trial displayed better benefits of TACE plus lenvatinib than TACE monotherapy, which means TKIs administration after TACE procedure may improve the efficacy of TACE. The rationale is clear to conduct ICIs treatment on the basis of inflammatory response and enhanced immunity after TACE.

Compared with monotherapy group, worse liver function, infusion-related reaction, proteinuria, pain and Hand-foot syndrome were observed in combination group. Fortunately, all adverse events were controlled and manageable with dosage reduction. Lower incidence rate of grade 3 or higher AEs occurred than those reported in IMbrave150, ORIENT-32 and CARES-310 trials (21.6% vs 63%, 21.6% vs 56% and 21.6% vs 71%). [21, 36, 37]Grade 3 or higher AEs in our study is similar to other real word studies (16.7% in CHANCE2211 and 15.8% in CHANCE2201). [23, 24] Safety is one of challenges in combination therapy. Collectively, TACE + TKIs + ICIs therapy had acceptable safety and improved the OS and PFS of patients.

There were several limitations in this study. Firstly, this was a retrospectively designed study, which may lead to potential selection bias. Besides, the sample size of the study is limited. The conclusions drawn from this study should be verified in better-designed prospective studies. Thirdly, a group of patients taking TKIs before TACE but accepting ICIs therapy after TACE was lacking, since several studies found that pre-treatment with TKIs before TACE can normalize tumor vessels and suppress VEGF, which may lead to a homogeneous distribution of lipiodol mixed anticancer drugs in the tumors [15, 36, 37]. This might be an issue to be further explored. Additionally, another limitation of our study was a dropout rate of approximately 10%, but without different significance between the two groups. We had tried our best to make that within a statistically reasonable range, but we need research with lower or even without dropout to improve the reliability of the results in the future. Finally, although we used PSM to minimize bias as much as possible, the diversity of TKIs and ICIs can still lead to biased results. It makes sense that similar results in TACE plus mono-TKI and mono-ICI but more rigorously prospective studies needed to confirm this.

In conclusion, our retrospective data indicated that TACE + TKIs + ICIs seemed to have considerable efficacy in patients with unresectable HCC compared to TACE monotherapy. Specially, “TKIs + ICIs” co-treatment within 3 months after the first TACE procedure might be a better medication strategy to achieve better clinical outcomes.

### Supplementary Information

**Additional file 1: Table S1.** Univariable and multivariable Cox regression analysis of baseline variables affecting OS. **Table S2**. The final status of patients after matching. **Figure S1.** Predictors of PD rate after matching.

## Data Availability

The datasets generated and/or analyzed during the current study are available from the corresponding author on reasonable request.

## Abbreviations

LC: Liver cancer
HCC: Hepatocellular carcinoma
TACE: Transarterial Chemoembolization
PSM: Propensity score matching
BCLC: Barcelona Clinic Liver Cancer
CNLC: China Liver Cancer Clinical
TME: Tumor microenvironment
ICIs: Immune checkpoint inhibitors
VEGF: Vascular endothelial growth factor
TKIs: Tyrosine kinase inhibitors
OS: Overall survival
PFS: Progression free survival
ORR: Objective response rate
DCR: Disease control rate
CR: Complete response
PR: Partial response
SD: Stable disease
PD: Progressive disease
AEs: Adverse events
