# Supplementary material for: Transarterial chemoembolization with/without immune checkpoint inhibitors plus tyrosine kinase inhibitors for unresectable hepatocellular carcinoma: a single center, propensity score matching real-world study
Source: Discov Oncol. 2024 Mar 9;15:68. doi: 10.1007/s12672-024-00917-1 (PMC10924872; doi:10.1007/s12672-024-00917-1)
Supplement: Supplementary file 1 — Additional file 1: Table S1. Univariable and multivariable Cox regression analysis of baseline variables affecting OS. Table S2. The final status of patients after matching. Figure S1. Predictors of PD rate after matching. [file 12672_2024_917_MOESM1_ESM.zip › New folder/Supplementary Table 1 Predictors of OS before matching.docx]

**Table 3** Univariate and multivariate Cox regression analysis of baseline variables affecting OS

|  | **Univariate** | | | **Multivariate** | | |
| --- | --- | --- | --- | --- | --- | --- |
|  | **HR** | **95% CI** | **P Value** | **HR** | **95% CI** | **P Value** |
| Age | 1.001 | 0.991-1.011 | 0.860 |  |  |  |
| Gender: M/ F | 0.986 | 0.669-1.452 | 0.941 |  |  |  |
| BCLC: B/C | 1.728 | 1.349-2.212 | <0.001 | 1.193 | 0.722-1.972 | 0.492 |
| ECOG performance: 0/1 | 0.999 | 0.729-1.367 | 0.993 |  |  |  |
| Tumor number: <3/≥3 | 0.729 | 0.550-0.966 | 0.028 | 0.720 | 0.540-0.960 | 0.025 |
| Tumor Size: <7cm/≥7cm | 1.046 | 0.816-1.340 | 0.723 |  |  |  |
| Embolus: Absent/Present | 1.655 | 1.277-2.169 | <0.001 | 1.223 | 0.759-1.971 | 0.407 |
| Extrahepatic metastasis: Absent/Present | 1.697 | 1.291-2.230 | <0.001 | 1.507 | 1.074-2.114 | 0.018 |
| AFP (ng/ml): <400/≥400 | 1.302 | 0.994-1.704 | 0.055 | 1.232 | 0.935-1.662 | 0.139 |
| Child-pugh grade: A/B | 0.942 | 0.673-1.319 | 0.729 |  |  |  |
| Group: TACE/TACE+TKIs+ICIs | 0.666 | 0.507-0.876 | 0.004 | 0.673 | 0.511-0.887 | 0.005 |

Note.—**OS**, Overall Sruvival; **ECOG**, Eastern Cooperative Oncology Group.
