# Supplementary material for: Transarterial chemoembolization with/without immune checkpoint inhibitors plus tyrosine kinase inhibitors for unresectable hepatocellular carcinoma: a single center, propensity score matching real-world study
Source: Discov Oncol. 2024 Mar 9;15:68. doi: 10.1007/s12672-024-00917-1 (PMC10924872; doi:10.1007/s12672-024-00917-1)
Supplement: Supplementary file 1 — Additional file 1: Table S1. Univariable and multivariable Cox regression analysis of baseline variables affecting OS. Table S2. The final status of patients after matching. Figure S1. Predictors of PD rate after matching. [file 12672_2024_917_MOESM1_ESM.zip › New folder/Supplementary Table2.docx]

Table 2 The final status between the two groups

|  |  | Group | | Total | *P* value |
| --- | --- | --- | --- | --- | --- |
|  |  | TACE | TACE+TKIs+ICIs |  |  |
| Status | Alive | 107(38.50%) | 66(47.5%) | 173(41.5%) | 0.084 |
|  | Died | 150(54.00%) | 59(42.40%) | 209(50.10%) |  |
|  | lost | 21(7.60%) | 14(10.10%) | 35(8.40%) |  |
| Total |  | 278(100%) | 139(100%) | 417(100%) |  |
