# Supplementary figures and images for: Transarterial chemoembolization with/without immune checkpoint inhibitors plus tyrosine kinase inhibitors for unresectable hepatocellular carcinoma: a single center, propensity score matching real-world study
Source: Discov Oncol. 2024 Mar 9;15:68. doi: 10.1007/s12672-024-00917-1 (PMC10924872; doi:10.1007/s12672-024-00917-1)

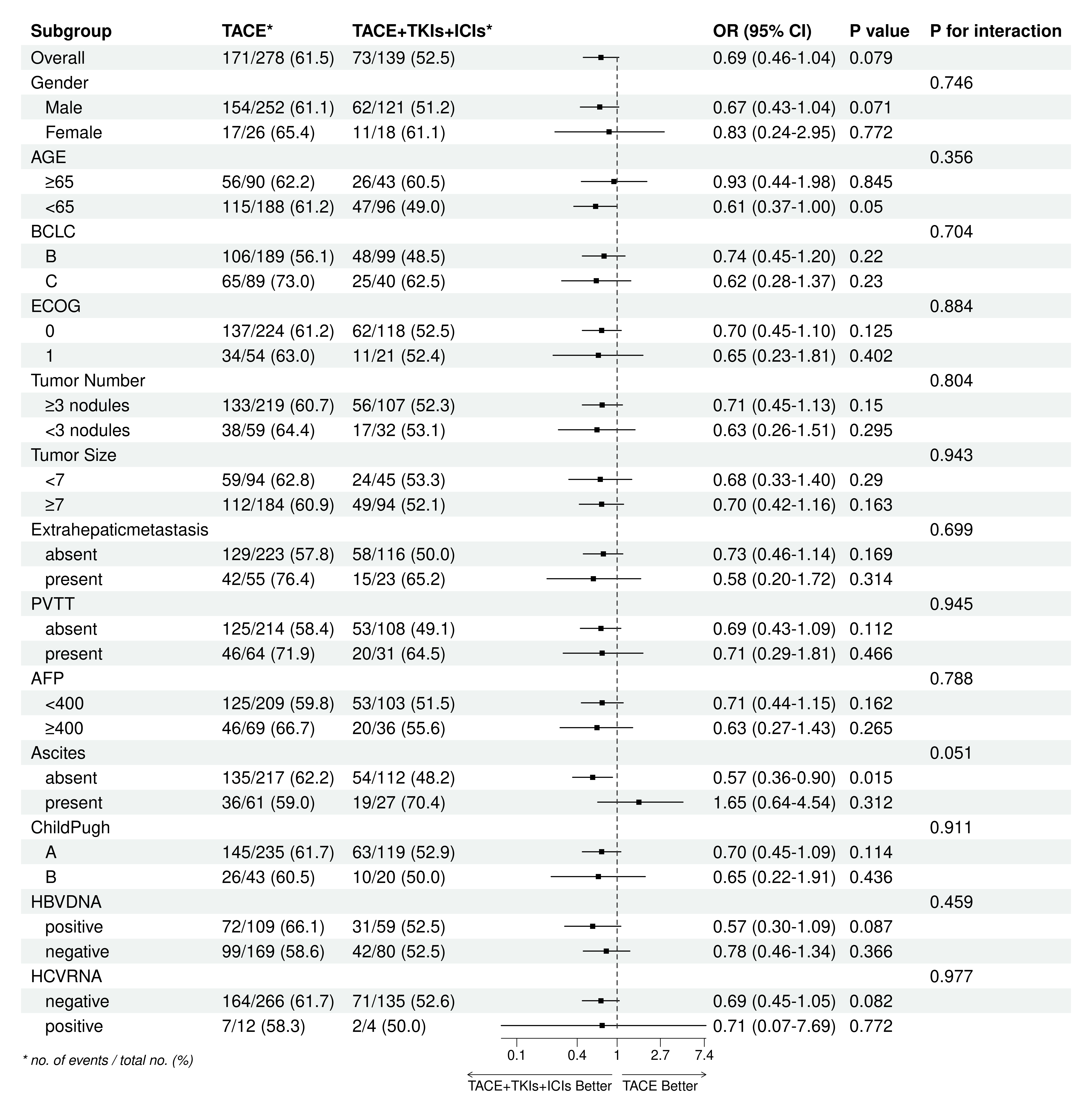

Supplement: Supplementary file 1 — Additional file 1: Table S1. Univariable and multivariable Cox regression analysis of baseline variables affecting OS. Table S2. The final status of patients after matching. Figure S1. Predictors of PD rate after matching. [file 12672_2024_917_MOESM1_ESM.zip › New folder/Supplementary Figure 1.png]
